# Clinical Notes and Reflections

**Published:** 1895-02

**Authors:** Jos. Fitz Mathew

**Affiliations:** Victoria, Dauphin Co., Pa.


### CLINICAL NOTES AND REFLECTIONS.

JOS. FITZ MATHEW, M. D., VICTORIA, DAUPHIN CO., PA.

Eczema rubrum. Man aged forty years, farm laborer, subject to boils. Face and frontal region swelled, inflamed and much disfigured; shooting pains in dimness and lachrymation of eyes; with dull frontal headache; intolerable burning itching of inflamed surface; worse at night and causing sleeplessness; acrid discharge has removed hair from eyebrows, frontal and temporal regions: ℞. Nat-mur.$^{2c}$ (Dunham). A few doses promptly relieved. As the face began to clear off, a large boil began to form on the leg. ℞. Sac-lac. with simple dressing. Boil discharged and healed. Patient is entirely free from eruption.

Acute periostites of the tibia.

Boy aged ten years; strumous family; brother died of "white swelling" of knee joint. A hot, reddened œdematous swelling over tarsal extremity of the tibia; very sensitive to touch. Dry tongue; rigors; temperature, 104.5°; pulse, 120. Excruciating pain, extorting screams, with periodical aggravations; worse in the warm bed, at night and toward morning. Exciting cause not apparent: ℞. Mezerium$^{2c}$ (Dunham); horizontal position; applications of lint dipped in mucilage of slippery-elm. Prompt relief of pain; temperature reduced to 102°; stop medicine. Watched carefully for fluctuation; temperature, normal. Effusion beneath periosteum evidently re-absorbed. Later, small superficial abscess formed, and discharged, then healed ; perfect recovery. From the severe nature of this case, and very unfavorable family history, it is evident that without the splendid action of Mezereum, a sub-periosteal abscess, with all its sequelæ, could not have been avoided.

The tenacity with which certain practitioners of Homœopathy, especially veterinarians, with whom it is a rule to which I am unaware of an exception—adhere to the idea that the larger the organism, the larger or more appreciable must be the dose or potency employed is remarkable. Allopathically, the dose of crude drugs must, in a measure, be governed by this rule ; although the superior efficiency of small doses of Quinia-sulph., for instance, was long ago demonstrated in the treatment of malarial fevers by army surgeons in India. Homœopathically —making allowance for the greater or lesser susceptibility and reactive powers of certain nervous organizations—it appears to to me that the size of an organism, biped or quadruped, should not determine the dose, and that a potency which will act upon a child is equally efficient for an adult, for a horse, and "why not" for an elephant, if we can get the simillimum.

Many allopaths admit the efficiency of homœopathic medicines in cases of infants and children, but deny their utility in those of adults. They have in many cases been forced to invite the use of homœopathic medicines in cases of young children because they dare not administer their *very appreciable* drugs. They know that analysis fails to reveal the presence of the drug in our attenuations, except in the very low ones, and that its action is neither mechanical or chemical but dynamic, and must act directly upon the nervous system through the papillæ of the tongue and lingual nerve. "Can they demonstrate that there is any change in these functions in the adult by which this dynamic power is inhibited?"

It is certain that there are many allopaths who are not so illogical as to doubt the equal efficiency of our potencies upon adult organisms, but they are not prepared for the self-sacrifice of an apostate, and thus permit their patients to believe that while Homœopathy may be advisable for children it is impotent for adults. I was once a resident in the family of an allopathic physician, whose wife had suffered excruciating pain from otalgia, for two days in spite of all remedies applied by her husband, and I was invited to prescribe. The simillimum was perfect for Pulsatilla. I drew the doctor's attention to this fact, and gave Puls.$^{2c}$, and in fifteen minutes the patient was relieved of all pain. The doctor subsequently made a few experiments for himself, which resulted in his being expelled from his society.

During a summer's outing on the Pacific Coast I was introduced to an allopath who, not knowing me to be a practitioner of Homœopathy, invited my assistance in the case of a little child with a severe case of pneumonia.

"Doctor, I am a homœopath."

"Oh! well; so much the better."

We saw the little patient. Ant-tart. was indicated, but I had left my pocket case in camp twenty miles off. We went to the drug store, got a grain of Ant-tart. potentized it, and it was administered by the doctor as I directed, stopping the medicine on signs of improvement.

I subsequently met Dr. ———, who informed me that the child, *which he had "given up,"* made a good recovery. Had the patient been an adult I should not have been invited to prescribe.

In the treatment of the diseases of horses I have found the higher potencies act most efficiently, when I have been able to get the simillimum, which, however, is often very difficult to get, the symptoms presented being only objective. Amongst several similar cases I select the following:

Phrenitis—Mad Staggers.

Dull, stupid; vacant expression of the eyes; staggering movements. Suddenly the eyes open; there is a wild, vacant stare, with dilated pupils; dilated nostrils and heaving flanks. The animal dashes about furiously; it is dangerous to go near him. Delirium supervenes; he falls on his side and lies unconscious, with clenched teeth and twitching lips. At this stage I approached and gave a few teaspoonfuls of a solution of a few pellets of Dunham's 2 C. Bell., running it in onto the tongue through the teeth—a glass syringe is best—at frequent intervals. Medicine was stopped upon signs of improvement, and in a few hours the horse was eating hay at the stack.

